# Neuronal and psychological underpinnings of pathological gambling

**DOI:** 10.3389/fnbeh.2014.00230

**Published:** 2014-07-01

**Authors:** Bryan F. Singer, Patrick Anselme, Mike J. F. Robinson, Paul Vezina

**Affiliations:** ^1^Department of Psychology, The University of MichiganAnn Arbor, MI, USA; ^2^Department of Psychology, The University of LiègeLiège, Belgium; ^3^Psychology Department, Wesleyan UniversityMiddletown, CT, USA; ^4^Department of Psychiatry and Behavioral Neuroscience, The University of ChicagoChicago, IL, USA

**Keywords:** dopamine, gambling, reward, uncertainty, addiction, ventral striatum, stress, conditioning (psychology)

Although pathological gambling (PG) is a prevalent disease, its neurobiological and psychological underpinnings are not well characterized. As legal gambling increases in prominence in a growing number of casinos as well as on the internet, the potential for a rise in PG diagnoses warrants investigation of the disorder. The recent reclassification of PG as a behavioral addiction in the DSM-5 raises the possibility that similar cognitive and motivational phenotypes may underlie both gambling and substance use disorders. Indeed, in this Research Topic, Zack et al. (2014) tested the hypothesis that exposure to reward unpredictability can recruit brain dopamine (DA) systems in a similar way to chronic exposure to drugs of abuse (see also Singer et al., 2012). Over the years a variety of models have proposed that alterations in DA signaling may mediate the transition from drug use to dependence; similarly, the hypothesis that aberrant DA responses may influence the transition from recreational, to problematic, and finally PG has only recently begun to be tested. The collection of articles in this Research Topic highlights the complexity of PG and posits several theories of how dopaminergic signaling may contribute to behavioral maladaptations that contribute to PG.

In this Research Topic, Paglieri et al. (2014) report a growing incidence of PG with a lack of effective treatments. As described by Goudriaan et al. (2014) (this Research Topic), PG is thought to result from “diminished cognitive control over the urge to engage in addictive behaviors” that manifests in the inability to control desire to gamble despite negative consequences. PG is characterized by several cognitive dysfunctions, including increased impulsivity and cognitive interference. Similar to drug addictions, gambling behavior is powerfully modulated by exposure to gambling-related conditioned stimuli. In this Research Topic both Anselme and Robinson (2013) as well as Linnet (2014) describe the supporting role of gambling-related cues in this behavioral addiction. Anselme and Robinson (2013) present a series of findings suggesting that surprising non-rewards enhance incentive salience attribution to conditioned cues in conditioning procedures as well as during gambling episodes. They discuss a possible evolutionary origin of this counterintuitive process. Linnet (2014) reviews the contribution of DA signaling to incentive salience and reward prediction. Noting the research demonstrating brain activation during gambling tasks despite the possibility of a loss, he suggests a role for DA dysfunction in reward “wanting” and anticipation.

Ventral striatal activation is thought to be critical for the attribution of incentive salience to reward-related cues. In this Research Topic, Lawrence and Brooks (2014) found that healthy individuals who are more likely to display disinhibitory personality traits, such as financial extravagance and irresponsibility, show increased capacity for ventral striatal DA synthesis. Thus it is possible that individual variation in DA signaling due to genetics or environmental factors may influence PG. Porchet et al. (2013) (this Research Topic) also investigated whether physiological and cognitive responses observed during the performance of gambling tasks could be altered in recreational gamblers with pharmacological manipulations. As the commentary from Zack (2013) suggests, the Porchet et al. (2013) results may reflect important differences in neurobiological function between recreational and pathological gamblers. This hypothesis, along with the results of Lawrence and Brooks (2014) demonstrating increased DA capacity in individuals thought to be more prone to gambling, illustrates the complexity of PG as a disease and the need to sample different populations with different techniques and behavioral tasks.

Two papers in this Research Topic suggest a role for cortisol in modulating incentive motivation in the ventral striatum. Li et al. (2014) demonstrate an imbalanced sensitivity to monetary vs. non-monetary incentives in the ventral striatum of pathological gamblers. They show that cortisol levels in PG positively correlate with ventral striatal responses to monetary cues. van den Bos et al. (2013) provide further evidence for the importance of cortisol by highlighting the strong positive correlation observed in men between salivary cortisol levels and risk-taking measures. This was a significant contrast to the weak negative correlation seen in women. Their findings highlight important gender differences in how stress hormones affect risky-decision making, and by extension, the role of stress in gambling.

In this Research Topic, Clark and Dagher (2014) provide a review of the literature investigating the relationship between DA agonists and impulse control disorders in Parkinson's patients, and how this relates to potential gains and losses within a decision-making framework. They provide the beginnings of a hypothetical model of how DA agonist treatments affect value and risk assessments. While a variety of research suggests that dopaminergic treatments for Parkinson's disease may affect PG, few have probed whether individuals with Huntington's disease (HD) display gambling-related phenotypes. Kalkhoven et al. (2014) (this Research Topic) show that HD patients exhibit symptoms of behavioral disinhibition similar to those observed in PG. However, HD patients do not typically develop problem gambling. Based on neurobehavioral evidence, these authors suggest why HD patients are unlikely to start gambling but have a higher chance of developing PG if they encounter a situation that promotes such behavior.

The investigation of neural mechanisms underlying PG is currently at an early stage. As emphasized by Potenza (2013) in this Research Topic, while previous research and the present findings suggest that DA may underlie gambling-related behaviors, other neurotransmitters and signaling pathways may also play vital roles in the emergence of the disease. Individual variation in PG populations (e.g., differing levels of impulsivity, compulsivity, decision making, and DA pathology) has produced discrepancies in the PG literature, warranting a systematic approach to investigating the disease in the future. Paglieri et al. (2014) also suggest the need for greater methodological integration of animal studies (rodents and primates) to better understand the mechanisms underlying PG. In particular, Tedford et al. (2014) note in this Research Topic that gambling activity involves costs/benefits decision-making and that intracranial self-stimulation provides experimental advantages over traditional reinforcement methods used to model PG in animals. Finally, Paglieri et al. (2014) suggest that computational modeling, already used to account for other psychiatric diseases, might be applied to PG as well. Taken together, this collection of articles suggests novel avenues for future research of PG to improve treatment options for the disease.

## Conflict of interest statement

The authors declare that the research was conducted in the absence of any commercial or financial relationships that could be construed as a potential conflict of interest.
